# Determining Sonication Effect on *E. coli* in Liquid Egg, Egg Yolk and Albumen and Inspecting Structural Property Changes by Near-Infrared Spectra

**DOI:** 10.3390/s21020398

**Published:** 2021-01-08

**Authors:** David Nagy, Jozsef Felfoldi, Andrea Taczmanne Bruckner, Csilla Mohacsi-Farkas, Zsanett Bodor, Istvan Kertesz, Csaba Nemeth, Viktoria Zsom-Muha

**Affiliations:** 1 Department of Physics and Control, Faculty of Food Science, Szent István University, 1118 Budapest, Hungary; Felfoldi.Jozsef@szie.hu (J.F.); Bodor.Zsanett@hallgato.uni-szie.hu (Z.B.); Kertesz.Istvan@szie.hu (I.K.); 2 Department of Microbiology and Biotechnology, Faculty of Food Science, Szent István University, 1118 Budapest, Hungary; Taczmanne.Bruckner.Andrea.Erzsebet@szie.hu (A.T.B.); Mohacsine.Farkas.Csilla@szie.hu (C.M.-F.); 3 Capriovus Kft., 2317 Szigetcsép, Hungary; nemeth.csaba@capriovus.hu

**Keywords:** ultrasound, egg, yolk, albumen, NIR, *E. coli*, aquaphotomics

## Abstract

In this study, liquid egg, albumen, and egg yolk were artificially inoculated with *E. coli.* Ultrasound equipment (20/40 kHz, 180/300 W; 30/45/60 min) with a circulation cooling system was used to lower the colony forming units (CFU) of *E. coli* samples. Frequency, absorbed power, energy dose, and duration of sonication showed a significant impact on *E. coli* with 0.5 log CFU/mL in albumen, 0.7 log CFU/mL in yolk and 0.5 log CFU/mL decrease at 40 kHz and 6.9 W absorbed power level. Significant linear correlation (*p* < 0.001) was observed between the energy dose of sonication and the decrease of *E. coli*. The results showed that sonication can be a useful tool as a supplementary method to reduce the number of microorganism in egg products. With near-infrared (NIR) spectra analysis we were able to detect the structural changes of the egg samples, due to ultrasonic treatment. Principal component analysis (PCA) showed that sonication can alter C–H, C–N, –OH and N–H bonds in egg. The aquagrams showed that sonication can alter the properties of H_2_O structure in egg products. The observed data showed that the absorbance of free water (1412 nm), water molecules with one (1440 nm), two (1462 nm), three (1472 nm) and four (1488 nm) hydrogen bonds, water solvation shell (1452 nm) and strongly bonded water (1512 nm) of the egg samples have been changed during ultrasonic treatment.

## 1. Introduction

Proteins are essential for human nutrition and among a wide variety of sources, egg is one of the best, which has numerous beneficial properties [1]. In the food industry it is also a widely used ingredient for different purposes.

Hen egg yolk consists of approximately 50% water, 30% lipids and 16% protein [2]. Due to its nutritional, organoleptic and functional properties, this is a widely used ingredient in various food products [3].

Egg white is a viscous biological fluid, which has antibacterial properties due to its physicochemical characteristics and antimicrobial proteins. Water in albumen represents 88.5% and proteins account for ~10% of composition. It contains about 40 different proteins, such as ovalbumin, lysozyme, ovomucoid, ovotransferrin. Ovalbumin, which constitutes about 54% of the total egg white protein, is mainly responsible for gelation [4,5,6,7].

Liquid egg is widely used to manufacture processed foods. However, the shelf life of these liquid products is quite low and in order to extend the shelf life they go through different processes. During industrial thermal processing egg proteins can be altered, leading to an undesirable functionality loss [8]. The coagulation of proteins at higher temperatures, also limits the pasteurization of egg to lower temperatures and longer duration [2,9]. This limitation can cause microbiological hazards, such as *Escherichia coli* contamination [10].

Therefore, there is a growing demand for more environmentally friendly technologies for food processing which can provide various functions in food quality and stability [11,12,13].

Many studies deal with the topic of using non-thermal or minimal processing technologies in an attempt to target microorganisms in foods, causing them to be more susceptible to other non-thermal processes or decrease the number of cells [14,15,16,17]. This emerging interest in using non-thermal technologies such as ultrasound to inactivate microorganisms in foods has led to many studies to investigate their efficacy and mechanisms of inactivation. These studies report that sonication alone or combined with other processes have a high potential to replace or assist traditional thermal processing methods and consider necessity of further research [18,19].

To measure the efficiency of minimal processing on structure and quality, in many studies scientists use non-destructive measurement techniques. There are many non-destructive techniques that can be used to measure quality properties of food such as electronic nose [20,21], ultrasound [22], near-infrared spectroscopy [23,24], ultraviolet-visible spectroscopy [25] and hyperspectral imaging [26].

Near-infrared spectroscopy (NIR) is able to analyze solid and liquid samples with no or minor pretreatment and it can be implemented in continuous methodologies [27]. Moreover, it can detect multiple chemical and structural compounds. The spectra itself corresponds to overtones and combinations of the fundamental molecular vibrations, however coupled with chemometrics it makes it possible to extract valuable information regarding the composition [28]. Furthermore, analysis of the NIR spectrum with aquaphotomics reveals information about covalent OH and hydrogen bonds. This method allows the structural changes, interactions and conformations in the contained water to be described by the absorption bands that are related to the overtones, vibrations and combinations of stretching of –OH [29,30].

The objective of this study is to determine the effect of ultrasound on microbiological contamination, particularly *E. coli* in egg samples. A further aim of this study is to investigate the sonication-caused structural changes with NIR spectrum analysis.

## 2. Materials and Methods

### 2.1. Materials

Three types of egg products such as whole liquid egg, egg yolk and egg white were investigated, supplied by Capriovus Kft. (Szigetcsép, Hungary). The products are made from “A” classified (as determined in 589/2008/EC regulation) homogenized and pasteurized fresh hen eggs. The samples were stored at 0–4 °C in 1 L plastic jugs before the dilution and measurements. Before every measurement the content of each individual jug was shaken, then the samples were poured into a bowl and gently mixed together.

### 2.2. Ultrasonic Treatment

The effect of ultrasound (US) treatment at different frequencies, power and duration on the physical, microbiological properties of egg was investigated using an ultrasonic bath (HBM Machines, Netherlands). The equipment is capable of delivering up to 300 W of power at 20/40 kHz frequency.

For sonication treatment the samples were prepared in two ways:

- for microbiological measurements 180 mL of the samples were poured into a 200 mL glass container after homogenization.
- for NIR measurements 18 mL of the samples were diluted with 162 mL of distilled water in order to obtain 10% (*w*/*w*) emulsions. We used the diluted samples in order to evaluate the NIR spectra from an aquaphotomics point of view, as in the case of aquaphotomics it is a common method to use solutions of water and samples [29,30].

**Figure 1:**
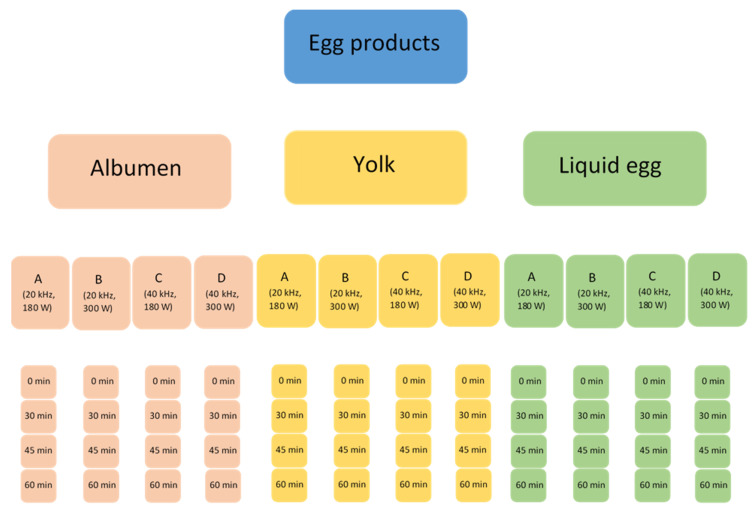
Treatment groups.

In both cases, the samples were separated into five further groups depending on the applied ultrasound parameters (Figure 1).

**Figure 2:**
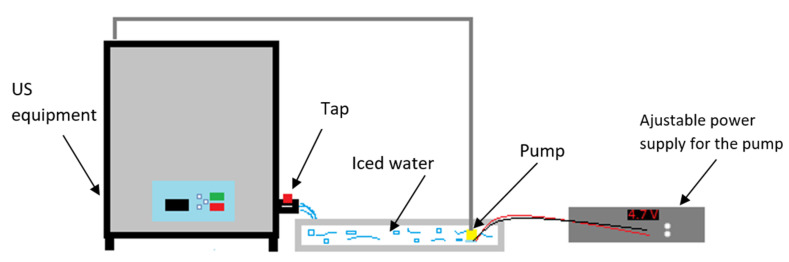
Schematic representation of the ultrasonic equipment with the circulating system.

These groups were separated into three more subgroups depending on the duration of the treatment (30 min, 45 min and 60 min). The ultrasound equipment was filled up with 16 L of tap water to ensure sonic conductivity and the containers were put into this media; 12 sealed, 200 mL glass containers were placed in the equipment at the same time. It was crucial to maintain a reasonably low temperature during the whole treatment, thus a circulation system with an external buffer was built (Figure 2). A tank, filled with iced water, was placed next to the ultrasound equipment and a submerged pump circulated this medium through the bath. This system is able to maintain the temperature at 18 ± 2 °C during the whole treatment. Untreated samples were subjected to the same temperature conditions as the sonicated ones.

To evaluate the absorbed ultrasonic power in the samples, preliminary experiments were carried out using distilled water. The water was poured into a 180 mL glass container. The lid of one container was drilled and a Pt100 temperature sensor was placed into the media through this hole. Twelve water samples were put into the equipment without the circulating system to measure the temperature change in the containers. Temperatures were measured at 180 W and 300 W power at 20 kHz and 40 kHz frequencies for 60 min. The measurements were carried out 4 times at every setup. The sample holder with the sensor was placed at different locations within the bath in every repeat. The absorbed power (W) was then determined calorimetrically according to the following equation [31]:

(1) $$P=mC_{p}{\left(\frac{dT}{dt}\right)}_{t=0}$$

where *m* is the mass (kg), *C_p_* (kJ·kg^−1^·K^−1^) is the specific heat capacity of distilled water and (*dT*/*dt*) is the rate of the change of temperature during the sonication process, determined by temperature changes in 30 s. The actual ultrasonic power dissipated in the liquid has been calculated to be 3.7 ± 0.1 and 6.9 ± 0.1 W, at 180 W and 300 W equipment power respectively for both frequencies.

The energy dose (J) of treatment was calculated by the multiplication of treatment duration (s) and the absorbed power (W).

### 2.3. Preparation of Artificial Inoculation

For microbiological measurements, 180 mL of samples were artificially inoculated with *Escherichia coli* (ATCC 25922). 180 µL of 1.5 × 10^8^ CFU/mL (determined by optical density) *E. coli* suspension was used for inoculation of the prepared samples.

**Figure 3:**
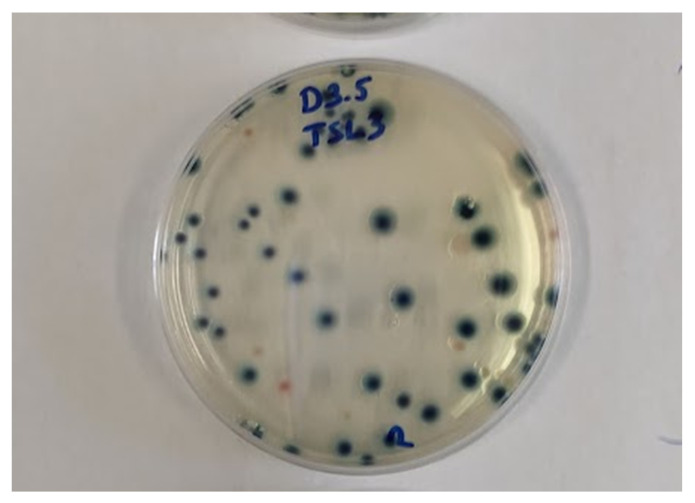
Blue discoloration of *E. coli* colonies on ChromoBio Coliform agar.

A selective and differential medium (ChromoBio COLIFORM, BioLab) was used to determine the colony forming units (CFU) of *E. coli* and distinguish *E. coli* colonies from other microorganism. This agar indicates the presence of *E. coli* with blue color (Figure 3).

The agar was prepared according to the manufacturer’s instructions.

A tenfold serial dilution was performed after sonication. 0.1 mL of the appropriate dilutions were transferred and spread on the surface of ChromoBio Coliform agar plates. Plates were incubated at 37 °C for 24–48 h. Control samples of liquid egg, yolk and albumen inoculated with *E. coli* before sonication were investigated in the same way. The control group was measured 3 times for liquid egg, yolk and albumen. Each sample was prepared in three replicates resulting 468 samples in total considering the different treatment groups. For visualization and analysis, the logarithm of the CFU was taken into account.

### 2.4. Near-Infrared Measurements

Near-infrared spectral analysis was carried out using a bench top (MetriNIR Research, Development and Service Co., Budapest, Hungary) spectrometer. The transflectance spectra were measured in the wavelength range of 740–1700 nm with the resolution of 2 nm. Spectra acquisition was performed using METRINIR measurement software v0.9.0.394 (Metrika Inc., Budapest, Hungary). Temperature can greatly influence the observed data, therefore it was crucial to maintain a constant temperature. To ensure this, a water cooled cuvette with a sample layer thickness of 0.4 mm was used. For NIR measurements, the 10 % (*w/w*) samples were taken into account. The samples were prepared in triplicate and randomly scanned by taking four consecutive scans of each, at 18 °C. That sums up to 540 samples for NIR measurements and from each sample four spectra were observed (2160 spectra).

### 2.5. Data Analysis

In order to detect whether the duration and treatment setups of sonication have a significant effect on microbiological properties, Two-way analysis of variance (ANOVA) was applied with Tukey honest significant difference (HSD) and Games–Howell tests at *p* < 0.05 significance level. Homogeneity of variance was tested by Levene’s test. The linear regression method was used to analyze the correlation between the energy dose of the treatment and the changes of log CFU/mL of *E. coli*.

In NIR data analysis the first step is to visualize the raw spectral data to detect outliers and to decide which pretreatment method is necessary to enhance the information relevant to the research. The Savitzky–Golay smoothing filter with second order polynomial with data frames of length 21 and no derivation was applied to the spectra, then for the baseline shift correction multiplicative scatter correction (MSC) was used.

The results of the NIR spectra of the samples were evaluated with principal component analysis (PCA). With PCA it is possible to break down large amounts of data into a few new variables, which contain the majority of variance of the original data [32]. It provides information on outliers, specific trends or whether there are groups in the data. In order to reduce the noise of the spectra, wavelength range of 950–1650 nm was taken into account. PCA models were built separately for liquid egg products and within these groups four models were calculated for treatment setups, which are summed up in 12 models.

Using PCA scores linear discriminant analysis (LDA) was performed to find a linear combination of features that may characterize the structure changes in the samples during sonication. The class variables were the durations of the different treatment setups (similarly as in PCA) in each liquid egg product, resulting in 12 models. A training dataset (recognition model) was used to build a prediction for the treatment duration effect. Two-third of the whole dataset was used to build this model. The accuracy of this predicting model was validated using threefold cross validation (data splitting and model building were performed three times), the average accuracy was calculated from the confusion tables obtained.

According to research of Tsenkova et al. [33,34] spectral range from 1300 to 1600 nm that contains the main 12 water matrix absorbance coordinates (WAMACs—water matrix coordinates) was adopted for our data analysis. Aquagram interpretation method was used to visualize the spectral pattern of our dataset. A classical aquagram is a radar chart displaying normalized absorbance at selected water bands.

Analysis of microbiological data was carried out by SPSS statistics 25 (IBM, Armonk, New York, NY, USA) and Microsoft Excel (Microsoft, Redmond, Washington, WA, USA) and NIR spectra analysis was done by RStudio 1.1.463 (RStudio Inc., Boston, MA, USA) with the “aquap2” [35] package.

## 3. Results

### 3.1. Microbiological Measurements

The results showed a slight reduction of *E. coli* in the egg samples respecting the treatment durations. The most prominent difference was observed at 60 min of treatment. At 40 kHz and 6.9 W of ultrasound treatment the reduction was 0.5 log CFU/mL in whole egg liquid, 0.7 log CFU/mL in yolk and 0.5 log CFU/mL in albumen.

The observed data showed that the control group was significantly (*p* < 0.005) distinguishable from every treated group. This means that sonication within the applied parameters has a slight, but significant effect on the survival of *E. coli* in liquid egg products. The Tukey test showed that the treatment setup and the duration of sonication have significant impact on *E. coli* log CFU/mL decrease alone. The interaction of these two properties were not significant.

The impact of sonication was detectable even on 20 kHz with 3.7 W power level. The analysis of the treatment setups compared to each other has also showed a significant difference in many cases.

The effect of treatment with 40 kHz and 6.9 W was significantly distinguishable from the albumen samples treated at 20 kHz and 6.9 W (*p* = 0.03) and 20 kHz and 3.7 W (*p* = 0.006), respectively.

Egg yolk showed a significant difference between samples treated at 20 kHz and 3.7 W and samples treated at 40 kHz and 3.7 W (*p* = 0.01), and between the samples sonicated at 20 kHz with the power of 3.7 W and 40 kHz with 6.9 W (*p* = 0.001), respectively.

The treatment setups showed no significant difference compared to each other in the case of egg liquid.

Durations of sonication also have a significant effect on *E. coli* decrease. In the case of 45 and 60 min of treatment Tukey test showed that, the impact of sonication was significant compared to the control group regardless to the treatment setup or the egg product. 30 min sonication showed a significant effect on liquid egg and albumen at every setup, but in the case of yolk the impact was not significant.

**Figure 4:**
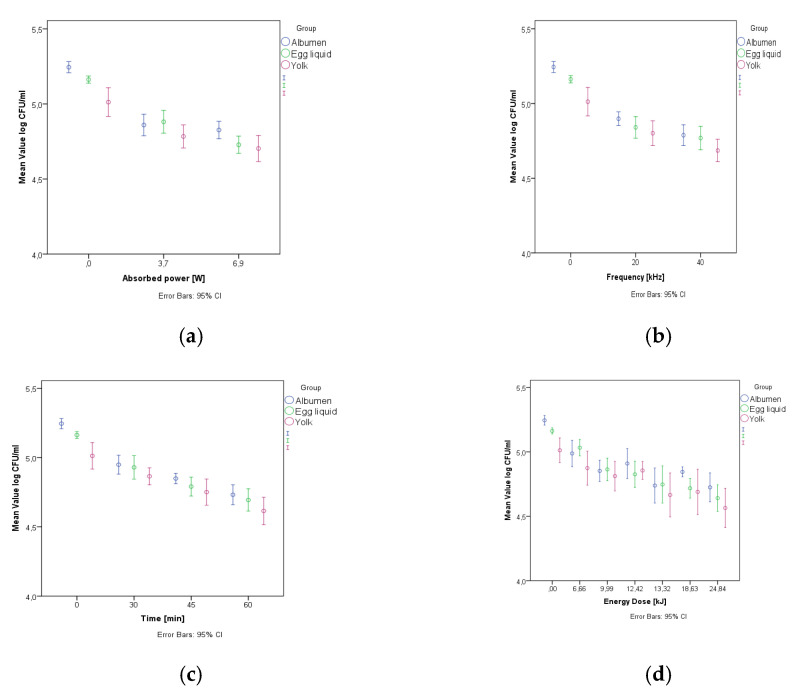
Log colony-forming unit (CFU)/mL decrease by absorbed power (**a**), frequency (**b**), time (**c**) and energy dose (**d**) for all samples.

*E coli* decrease was evaluated vs. absorbed power (W), frequency of sonication (kHz), duration (min) and the dose of treatment (kJ) (Figure 4).

The absorbed power levels showed a significant impact on *E. coli* count. The applied 3.7 and 6.9 W were sufficient to achieve a significant decrease compare to the control.

Frequency levels of 20 kHz and 40 kHz were significantly distinguishable from the control group. The 20 kHz and 40 kHz were also significantly different from each other (*p* = 0.002) according to the Games–Howell test.

Applied dose levels significantly differ from control group. The significant effect of energy dose of the sonication was detectable even on the lowest level (6.66 kJ). However, the groups close to each other were not significantly distinguishable from each other. The Games–Howell test showed that higher levels of dose were significantly distinguishable from lower levels of dose.

**Figure 5:**
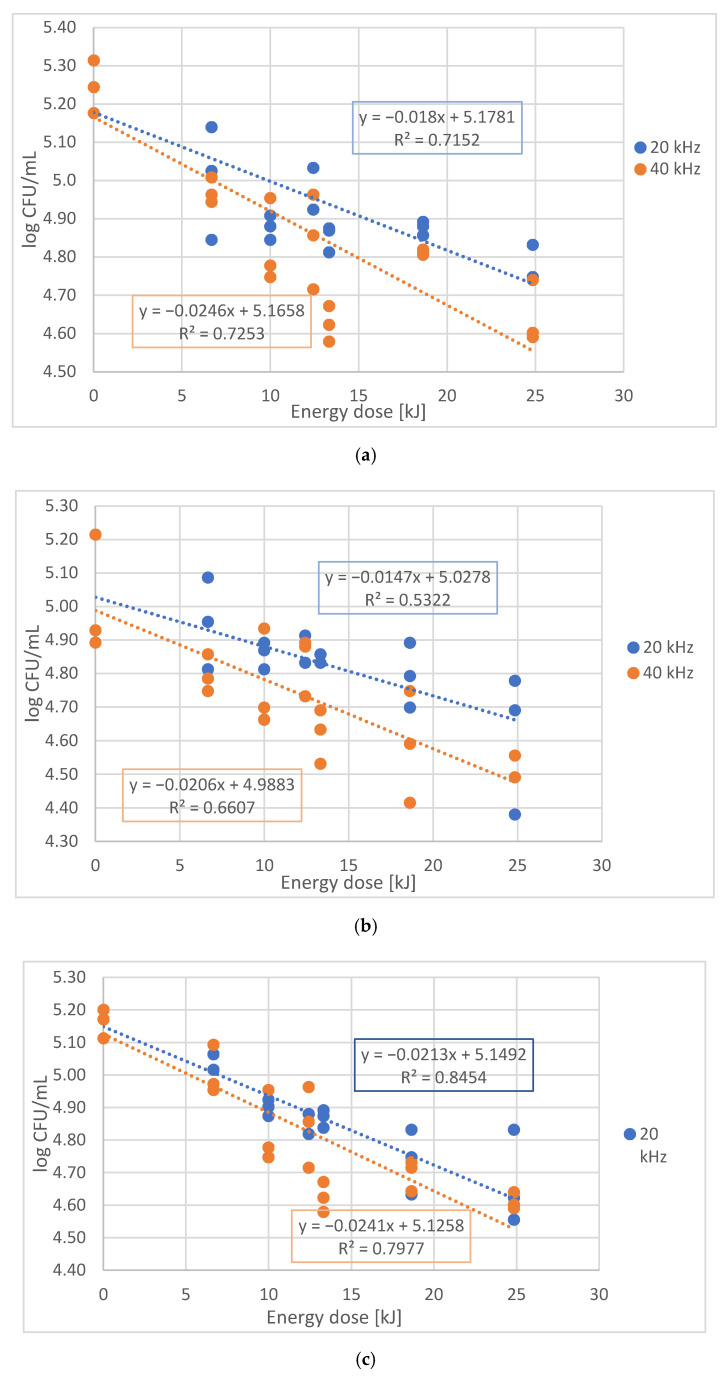
Linear regression of log CFU/mL (y) by the energy dose (x) of the treatment separately for 20 kHz and 40 kHz ((**a**) albumen, (**b**) yolk, (**c**) liquid egg).

Negative correlation was observed between the energy dose of sonication and the decrease of *E. coli (*Figure 5*)*. The slopes, R^2^, and F values of the models for all cases are summarized in Table 1.

Although, the parameters presented in Table 1, show a moderately strong correlation (due to the high variability of the microbiological measurement results), in all experiments significant linear model was found (*p* < 0.001) between the dose and the lethal effect. The highest energy dose of sonication with 24.48 kJ was able to reduce the *E. coli* by 0.5 log CFU/mL in whole egg liquid, 0.7 log CFU/mL in yolk and 0.5 log CFU/mL in albumen Although the difference between the effect of frequencies was not significant, we found that treatments at 20 kHz were systematically less effective than at 40 kHz.

We noted that treatments were less effective for yolk (the difference is not significant between the treated egg products).

### 3.2. Results of the Near-Infrared (NIR) Measurements

**Figure 6:**
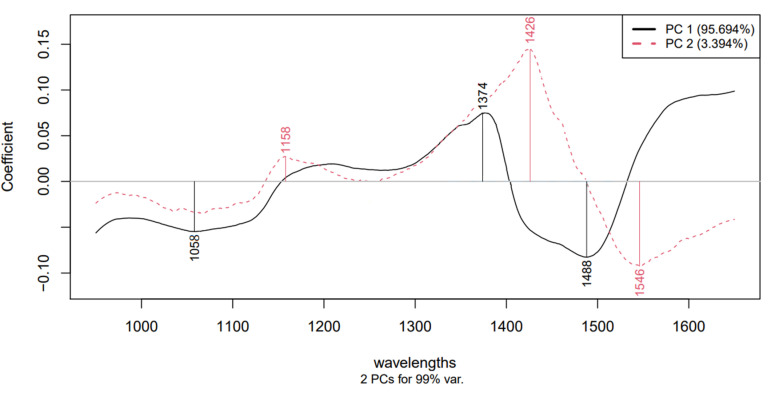
Loading plot of liquid egg treated at 20 kHz and 3.7 W.

The PCA models built using NIR spectra of egg samples show that the first two principal components describe the variance between 85% and 99% for all models. The higher duration was visually more separable. In particular, the 60 min treatment groups were distinguishable from lower duration groups through PC1 and PC2. The wavelengths that contributed to the formation of the first two principal components are listed in Table 2 based on the models. Figure 6 represents an example of the loading plots where the wavelengths highly contributing to the formation of PC1 and PC2 were acquired.

The exact molecules that changed are not determined, although there are wavelengths that related to –OH bonds in the range of 1300 to 1600 nm that can be analyzed further with aquaphotomics and the water molecule structures and conformations can be characterized.

**Figure 7:**
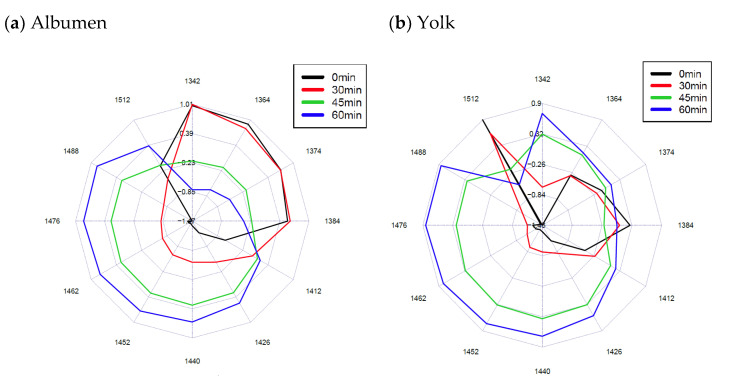

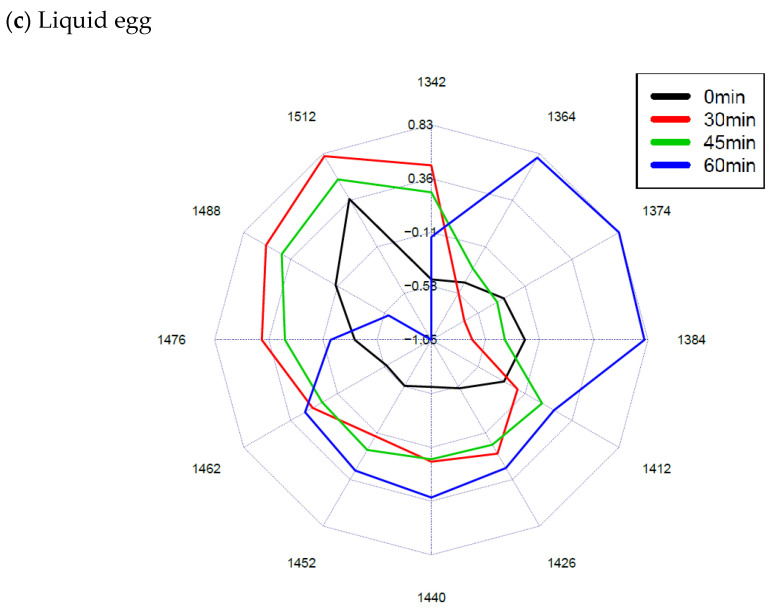
Aquagrams. Average values of normalized absorbance values of the water matrix coordinates of albumen (**a**), yolk (**b**) and liquid egg (**c**) treated at 40 kHz at 6.9 W.

To visualize WAMACs the raw spectral data from 1300 to 1600 nm were used to create aquagrams (Figure 7).

Aquagrams showed that sonication can alter the properties of H_2_O structure in egg products. There is an increasing trend in albumen and yolk at wavelengths 1412, 1426, 1440, 1452, 1462, 1488 nm especially at 40 kHz and 6.9 W according to the duration of sonication. A well traceable decreasing trend did not occur in the case of albumen at 1342, 1364 and 1374 nm. This decreasing trend did not occur in the case of yolk, but an increase was observed at 1342, 1364 and 1374 nm. Absorbance trend for liquid egg showed a different trend compared to the yolk and albumen. An increase in absorbance was observed at 1412, 1426, 1440, 1452, 1462 nm. The absorbance at 1364, 1374 and 1384 in the case of 60 min sonication showed an increase in liquid egg. These trends were also apparent with on the other treatment setups at a slightly lower tendency.

### 3.3. Linear Discriminant Analysis

The model built with LDA showed that sonication durations can be differentiated, especially in the case of 60 min of treatment. The accuracy of training and validated prediction models are shown in Table 3.

Classification results of the LDA models (Table 4) showed no overlapping between the control group and the 60 min treated groups in albumen, yolk and egg liquid in both training and validation models. These models shows that sonication duration and treatment setups have differentiating effect on liquid egg products. Sixty minutes of sonication was distinguishable from the control group in every case regardless of the treatment setup in both training and validation models. The samples treated for 45 min showed misclassification with the control group in the case of liquid egg at 40 kHz and 6.9 W treatment (5.02%) and treated at 20 kHz with 6.9 W (16.67%) and at yolk samples at treated at 40 kHz with 6.9 W (4.5%) and at 20 kHz with 6.9 W power (16.62%) in the training model, respectively. Based on the models 30 min of sonication overlapped in almost every case with the control group.

The validation model showed a slight overlap between 60 min and 45 min at 40 kHz with 6.9 W treatment (5.22%) and 20 kHz with 3.7 W treatment in albumen (5.21%), in liquid egg treated at 20 kHz and 6.9 W (21.77%) and yolk samples that are treated at 40 kHz with the power level 6.9 W (9.57%), respectively. In two cases the 60 min and 30 min groups showed an overlap in albumen treated at 20 kHz with 3.7 W (16.75%) and at 40 kHz with 6.9 W absorbed power (18.26%).

The 30 min treated samples were overlapping in almost every case with the untreated ones in both training and prediction models.

The overlapping occurring at higher durations may be caused by the applied power level.

## 4. Discussion

### 4.1. Microbiological Measurements

Frequency, absorbed power, energy dose, and duration of sonication showed significant impact on *E. coli* decrease. We observed 0.5 log CFU/mL in albumen, 0.7 log CFU/mL in yolk and 0.5 log CFU/mL at 40 kHz and a 6.9 W absorbed power level. Observed data show sonication alone was not able to reduce the *E. coli* at an acceptable quantity, especially at this level of power and duration. This confirms previously reported results by Inguglia et al. [36].

According to European regulation the acceptable level of *E. coli* contamination in egg products is between 1 to 2 log CFU/mL (2073/2005/EC). This level was not reached from the initial level of 5 log CFU/mL in our experimental design with the applied treatment setups, but extending the linear model we can assume that an about seven times higher dose of sonication would be able to reduce the *E. coli* by 5 log CFU/mL (although the standard deviation would be much higher).

Our results are in accordance with studies reporting that ultrasound as a combined treatment has a huge potential to inactivate microorganism [37,38,39].

The lethal effect of sonication on *E. coli* indicates that this process can be an efficient complementary technology to aid heat treatment for industrial purposes. Furthermore the experimental setting necessitated the application of a non-continuous process, but in an industrial setting a continuous process is preferred, which can allow more efficient utilization at an increased power level, therefore decreasing the required sonication time.

### 4.2. NIR Measurements

With near infrared spectrum analysis we were able to detect changes in liquid egg products caused by sonication. The models built based on principal components were able to detect the impact of different treatment setups and durations. With PCA analysis we obtained wavelengths that are related to molecular bonds that contribute to these changes.

The observed contributing wavelengths (Table 2) are related to different bonds in the liquid egg, yolk and albumen. Thus, it can be presumed that bonds that refer to these wavelengths are altered during the sonication process. Based on the research of Tsenkova et al., Muncan et al., Mayo et al. and Szigedi [33,34,40,41] the bonds associated with C–N valence vibrations are moderately strong for primary amines and are found in the range of 1040 to 1080 nm. The absorption bonds of secondary amines are of medium intensity around 1140 to 1180 nm. Typically, the valence and deformation vibrations of the C–C bonds are found in the range of 1100 to 1300 nm. The wavelength range 1300–1550 nm refers to the first harmonic range of the –OH valence vibration. The bonds for N–H deformation vibrations are strong for primary amines at the range between 1590 to 1650 nm. The absorption bonds of the deformation vibrations of secondary amines are of medium intensity in the range of 1550 to 1650 nm.

Based on the contributing wavelengths the C–N, C–C, –OH and N–H were altered during the sonication in almost every group.

Further investigation of water molecule structures and conformations at wavelength 1300 to 1600 nm was performed based on the research of Tsenkova et al. [33,34]. The observed data at 1412 nm showed that the free water absorbance of the albumen, yolk and liquid egg increased during ultrasonic treatment, while on the other hand the highly bonded water absorbance decrease based on the wavelength 1512 nm in yolk.

The changes at the wavelengths 1440, 1452, 1462, 1476 and 1488 nm show that the water molecules with one, two, three and four hydrogen bonds and water solvation shell absorbance have increased in egg products during the treatment. However, the trend in the case of liquid egg samples treated for 60 min at wavelengths 1476 and 1488 nm, that related to water molecules with three and four hydrogen bonds, was not verified by the aquagram.

The water solvation shell and symmetrical stretching vibration also showed an increased absorbance at the wavelengths 1364, 1374 nm in the case of liquid egg samples treated for 60 min.

In the presented research we have not aimed to determine the sonication effect on definite molecules and physical and techno-functional properties. The studies of Stefanivic et al., Xie et al. and Jovanovic et al. [42,43,44] report enhancement in foaming, emulsification properties and protein hydrolysis in egg products, due to sonication treatment, that may be traceable with near infrared spectral measurements as well. This highlights that our further investigations can be aimed at these property changes and a relationship between NIR spectral analysis.

## 5. Conclusions

Although sonication used at lower level of power had a significant effect on *Escherichia coli* degradation, the acceptable level was not reached. In an industrial environment there are more powerful and continuous methods of sonication, where the power absorbed by the samples can be much higher, therefore those setups have more of an effect on microbiological contamination. It is crucial to optimize the applied frequency, power and duration of treatment, because it can cause structural changes in eggs, as well. The NIR spectroscopy measurements showed that the duration of the treatments significantly affected the contained water molecules in egg. Models built by PCA scores showed that 60 min of treatment even at lower power level is 100% distinguishable from untreated samples. That indicates NIR spectroscopy can be a useful tool for monitoring the changes in egg products treated by ultrasound.

## Figures and Tables

**Table 1:** Slopes values, standard deviation (SD) of the slopes, F values and coefficient of determination (R^2^) values of the models.

Group	Frequency	Slope of the Model (log CFU/kJ)	SD of the Slope	F Value	R^2^ Values
Albumen	20 kHz	−0.018	0.002	55.238	0.7152
Albumen	40 kHz	−0.025	0.003	58.093	0.7253
Yolk	20 kHz	−0.015	0.004	18.486	0.5322
Yolk	40 kHz	−0.021	0.003	42.844	0.6607
Liquid egg	20 kHz	−0.021	0.002	120.311	0.8454
Liquid egg	40 kHz	−0.024	0.003	86.761	0.7997

**Table 2:** The contributing wavelengths for principal component analysis (PCA) based on loading values.

Egg Product	Treatment Setup	Wavelengths			
		**C–N**	**C–C**	**–OH**	**N–H**
Albumen	20 kHz, 3.7 W	1074	1194	1407, 1482, 1512	1620
	20 kHz, 6.9 W	1052, 1100	-	1412, 1508	1554
	40 kHz, 3.7 W	1078	1184	1384, 1462, 1512, 1548	-
	40 kHz, 6.9 W	1066	-	1342, 1412, 1440, 1513	1560
Yolk	20 kHz, 3.7 W	-	1214	1504	1660
	20 kHz, 6.9 W	1026, 1070	-	1374, 1502	-
	40 kHz, 3.7 W	1066	1206	1462, 1504	-
	40 kHz, 6.9 W	1060	1206	1384, 1452, 1534	-
Liquid egg	20 kHz, 3.7 W	1058, 1158	-	1374, 1426, 1488, 1546	-
	20 kHz, 6.9 W	1051, 1156	1208	1398, 1476, 1548	-
	40 kHz, 3.7 W	1060	-	1412, 1520	1616
	40 kHz, 6.9 W	1056	1210	1406,1492,1544	-

**Table 3:** The accuracy of the observed models.

Group	Treatment	Recognition	Prediction
Albumen	A	93.97%	83.33%
Yolk	A	87.29%	79.61%
Liquid egg	A	75.57%	64.04%
Albumen	B	100.0%	92.13%
Yolk	B	68.61%	66.35%
Liquid egg	B	90.80%	55.67%
Albumen	C	96.86%	86.07%
Yolk	C	90.09%	92.77%
Liquid egg	C	100.0%	88.38%
Albumen	D	89.03%	86.67%
Yolk	D	91.39%	61.46%
Liquid egg	D	91.40%	61.47%

**Table 4:** Classification table of the acquired linear discriminant analysis (LDA) models.

**Albumen**								
	**Prediction(%)**				**Validation(%)**			
Treatment A	0 min	30 min	45 min	60 min	0 min	30 min	45 min	60 min
0 min	**100**	0	0	0	**100**	16.75	0	0
30 min	0	**100**	0	0	0	**58.25**	0	0
45 min	0	0	**97.75**	21.86	0	8.25	**100**	24.95
60 min	0	0	2.25	**78.14**	0	16.75	0	**75.05**
Treatment B	0 min	30 min	45 min	60 min	0 min	30 min	45 min	60 min
0 min	**100**	0	0	0	**100**	0	21.04	0
30 min	0	**100**	0	0	0	**100**	5.22	0
45 min	0	0	**100**	0	0	0	**68.51**	0
60 min	0	0	0	**100**	0	0	5.22	**100**
Treatment C	0 min	30 min	45 min	60 min	0 min	30 min	45 min	60 min
0 min	**100**	12.55	0	0	**100**	16.75	0	0
30 min	0	**87.45**	0	0	0	**58.25**	0	0
45 min	0	0	**100**	0	0	8.25	**100**	24.95
60 min	0	0	0	**100**	0	16.75	0	**75.05**
Treatment D	0 min	30 min	45 min	60 min	0 min	30 min	45 min	60 min
0 min	**83.38**	9.14	0	0	**83.25**	8.99	0	0
30 min	16.62	**72.71**	0	0	16.75	**72.75**	0	0
45 min	0	0	**100**	0	0	0	**94.79**	4.12
60 min	0	18.14	0	**100**	0	18.26	5.21	**95.88**
**Yolk**								
	**Prediction(%)**				**Validation(%)**			
Treatment A	0 min	30 min	45 min	60 min	0 min	30 min	45 min	60 min
0 min	**79.12**	19.94	0	0	**75.00**	30.03	0	0
30 min	20.88	**70.01**	0	0	25.00	**60.06**	16.62	0
45 min	0	10.04	**100**	0	0	9.91	**83.38**	0
60 min	0	0	0	**100**	0	0	0	**100**
Treatment B	0 min	30 min	45 min	60 min	0 min	30 min	45 min	60 min
0 min	**50**	25	28.57	0	**58.25**	41.75	28.57	0
30 min	33.38	**62.5**	9.5	0	25	**50.00**	14.29	0
45 min	16.62	12.5	**61.93**	0	16.75	8.25	**57.14**	0
60 min	0	0	0	**100**	0	0	0	**100**
Treatment C	0 min	30 min	45 min	60 min	0 min	30 min	45 min	60 min
0 min	**91.62**	31.27	0	0	**91.75**	12.41	8.25	0
30 min	8.38	**68.73**	0	0	8.25	**87.59**	0	0
45 min	0	0	**100**	0	0	0	**91.75**	0
60 min	0	0	0	**100**	0	0	0	**100**
Treatment D	0 min	30 min	45 min	60 min	0 min	30 min	45 min	60 min
0 min	**81.86**	9.13	7.14	0	**63.66**	27.32	33.29	0
30 min	13.64	**90.87**	0	0	9.02	**63.66**	28.57	10.04
45 min	4.5	0	**92.86**	0	27.32	9.02	**28.57**	0
60 min	0	0	0	**100**	0	0	9.57	**89.96**
**Liquid Egg**								
	**Prediction (%)**				**Validation (%)**			
Treatment A	0 min	30 min	45 min	60 min	0 min	30 min	45 min	60 min
0 min	**75**	54.57	18.14	0	**83.25**	72.75	9.02	0
30 min	25	**45.43**	0	0	16.75	**18.26**	36.34	0
45 min	0	0	**81.86**	0	0	8.99	**54.64**	0
60 min	0	0	0	**100**	0	0	0	**100**
Treatment B	0 min	30 min	45 min	60 min	0 min	30 min	45 min	60 min
0 min	**77.83**	4.12	0	0	**55.67**	16.75	30.38	0
30 min	5.5	**87.5**	2.15	0	0	**58.25**	39.11	0
45 min	16.67	8.38	**97.85**	0	44.33	25	**8.74**	0
60 min	0	0	0	**100**	0	0	21.77	**100**
Treatment C	0 min	30 min	45 min	60 min	0 min	30 min	45 min	60 min
0 min	**100**	0	0	0	**90.98**	37.45	0	0
30 min	0	**100**	0	0	9.02	**62.55**	0	0
45 min	0	0	**100**	0	0	0	**100**	0
60 min	0	0	0	**100**	0	0	0	**100**
Treatment D	0 min	30 min	45 min	60 min	0 min	30 min	45 min	60 min
0 min	**37.52**	18.14	5.02	0	**37.59**	18.26	25.04	0
30 min	0	**31.79**	7.5	0	12.41	**36.24**	14.99	0
45 min	62.48	50.07	**87.48**	0	50	45.5	**59.97**	0
60 min	0	0	0	**100**	0	0	0	**100**

## Data Availability

The data presented in this study are available on request from the corresponding author. The data are not publicly available due to this research is still in progress.

## References

[1] Liu Y.-F., Oey I., Bremer P., Carne A., Silcock P. (2019). Modifying the Functional Properties of Egg Proteins Using Novel Processing Techniques: A Review. Compr. Rev. Food Sci. Food Saf..

[2] Wang R., Ma Y., Ma Z., Du Q., Zhao Y., Chi Y. (2020). Changes in gelation, aggregation and intermolecular forces in frozen-thawed egg yolks during freezing. Food Hydrocoll..

[3] Anton M. (2013). Egg yolk: Structures, functionalities and processes. J. Sci. Food Agric..

[4] Arzeni C., Perez O.E., Pilosof A.M.R. (2015). Power Ultrasound Assisted Design of Egg Albumin Nanoparticles. Food Biophys..

[5] Hammershoj M., Larsen L.B., Andersen A.B., Qvist K.B. (2002). Storage of shell eggs influences the albumen gelling properties. LWT Food Sci. Technol..

[6] Jun S., Yaoyao M., Hui J., Obadi M., Zhongwei C., Bin X. (2020). Effects of single- and dual-frequency ultrasound on the functionality of egg white protein. J. Food Eng..

[7] Karoui R., De Ketelaere B., Kemps B., Bamelis F., Mertens K., De Baerdemaeker J., Sun D.-W. (2009). Chapter 15—Eggs and Egg Products. Infrared Spectroscopy for Food Quality Analysis and Control.

[8] Monfort S., Manas P., Condon S., Raso J., Alvarez I. (2012). Physicochemical and functional properties of liquid whole egg treated by the application of Pulsed Electric Fields followed by heat in the presence of triethyl citrate. Food Res. Int..

[9] Chandrapala J., Zisu B., Palmer M., Kentish S., Ashokkumar M. (2011). Effects of ultrasound on the thermal and structural characteristics of proteins in reconstituted whey protein concentrate. Ultrason. Sonochem..

[10] Bell C., Kyriakides A., Blackburn C.W., McClure P.J. (2009). Pathogenic Escherichia coli. Foodborne Pathogens: Hazards, Risk Analysis And Control.

[11] Punidadas P., McKellar R.C. (1999). Selected physical properties of liquid egg products at pasteurization temperatures. J. Food Process. Preserv..

[12] Afraz M.T., Khan M.R., Roobab U., Noranizan M.A., Tiwari B.K., Rashid M.T., Inam-ur-Raheem M., Hashemi S.M.B., Aadil R.M. (2020). Impact of novel processing techniques on the functional properties of egg products and derivatives: A review. J. Food Process Eng..

[13] Fernández-Martin F., Fernández P., Carballo J., Colmenero F.J. (1997). Pressure/heat combinations on pork meat batters: Protein thermal behavior and product rheological properties. J. Agric. Food Chem..

[14] Paniagua-Martinez I., Ramirez-Martinez A., Serment-Moreno V., Rodrigues S., Ozuna C. (2018). Non-thermal Technologies as Alternative Methods for Saccharomyces cerevisiae Inactivation in Liquid Media: A Review. Food Bioprocess Technol..

[15] Lee D.-U. (2009). Effects of Combination Treatments of Nisin and High-intensity Ultrasound with High Pressure on the Functional Properties of Liquid Whole Egg. Food Sci. Biotechnol..

[16] Huang E., Mittal G.S., Griffiths M.W. (2006). Inactivation of Salmonella enteritidis in liquid whole egg using combination treatments of pulsed electric field, high pressure and ultrasound. Biosyst. Eng..

[17] Gómez-Sánchez D.L., Antonio-Gutiérrez O., López-Díaz A.S., Palou E., López-Malo A., Ramirez-Corona N. (2020). Performance of combined technologies for the inactivation of Saccharomyces cerevisiae and Escherichia coli in pomegranate juice: The effects of a continuous-flow UV-Microwave system. J. Food Process Eng..

[18] Huang G., Chen S., Dai C., Sun L., Sun W., Tang Y., Xiong F., He R., Ma H. (2017). Effects of ultrasound on microbial growth and enzyme activity. Ultrason. Sonochem..

[19] Kentish S., Feng H. (2014). Applications of Power Ultrasound in Food Processing. Annu. Rev. Food Sci. Technol..

[20] Kaya A., Keceli A.S., Catal C., Tekinerdogan B. (2020). Sensor Failure Tolerable Machine Learning-Based Food Quality Prediction Model. Sensors.

[21] Peris M., Escuder-Gilabert L. (2009). A 21st century technique for food control: Electronic noses. Anal. Chim. Acta.

[22] Awad T.S., Moharram H.A., Shaltout O.E., Asker D., Youssef M.M. (2012). Applications of ultrasound in analysis, processing and quality control of food: A review. Food Res. Int..

[23] Moon E.J., Kim Y., Xu Y., Na Y., Giaccia A.J., Lee J.H. (2020). Evaluation of Salmon, Tuna, and Beef Freshness Using a Portable Spectrometer. Sensors.

[24] Curto B., Moreno V., Garcia-Esteban J.A., Javier Blanco F., Gonzalez I., Vivar A., Revilla I. (2020). Accurate Prediction of Sensory Attributes of Cheese Using Near-Infrared Spectroscopy Based on Artificial Neural Network. Sensors.

[25] Hassoun A., Carpena M., Prieto M.A., Simal-Gandara J., Özogul F., Özogul Y., Özlem E.C., Gudjónsdóttir M., Barba F.J., Marti-Quijal F.J. (2020). Use of Spectroscopic Techniques to Monitor Changes in Food Quality during Application of Natural Preservatives: A Review. Antioxidants.

[26] Huang H., Liu L., Ngadi M.O. (2014). Recent Developments in Hyperspectral Imaging for Assessment of Food Quality and Safety. Sensors.

[27] Blanco M., Villarroya I. (2002). NIR spectroscopy: A rapid-response analytical tool. TrAC Trends Anal. Chem..

[28] Chen H., Tan C., Lin Z. (2019). Non-destructive identification of native egg by near-infrared spectroscopy and data driven-based class-modeling. Spectrochim. Acta Part A Mol. Biomol. Spectrosc..

[29] Bázár G., Kovacs Z., Tanaka M., Furukawa A., Nagai A., Osawa M., Itakura Y., Sugiyama H., Tsenkova R. (2015). Water revealed as molecular mirror when measuring low concentrations of sugar with near infrared light. Anal. Chim. Acta.

[30] Kovacs Z., Pollner B., Bazar G., Muncan J., Tsenkova R. (2020). A Novel Tool for Visualization of Water Molecular Structure and Its Changes, Expressed on the Scale of Temperature Influence. Molecules.

[31] Tiwari B.K., Mason T.J., Cullen P.J., Tiwari B.K., Valdramidis V.P. (2012). Ultrasound Processing of Fluid Foods. Novel Thermal and Non-Thermal Technologies for Fluid Foods.

[32] Barbin D.F., Badaro A.T., Honorato D.C.B., Ida E.Y., Shimokomaki M. (2020). Identification of turkey meat and processed products using near infrared spectroscopy. Food Control.

[33] Muncan J., Tsenkova R. (2019). Aquaphotomics-From Innovative Knowledge to Integrative Platform in Science and Technology. Molecules.

[34] Tsenkova R. (2009). Introduction Aquaphotomics: Dynamic spectroscopy of aqueous and biological systems describes peculiarities of water. J. Near Infrared Spectrosc..

[35] Kovacs Z., Pollner B. Aquaphotomics-Software R-Package “aquap2“. Proceedings of the Understanding Water in Biology 2nd International Symposium.

[36] Inguglia E.S., Tiwari B.K., Kerry J.P., Burgess C.M. (2018). Effects of high intensity ultrasound on the inactivation profiles of Escherichia coli K12 and Listeria innocua with salt and salt replacers. Ultrason. Sonochem..

[37] Patil S., Bourke P., Kelly B., Frias J.M., Cullen P.J. (2009). The effects of acid adaptation on Escherichia coli inactivation using power ultrasound. Innov. Food Sci. Emerg. Technol..

[38] De Sao Jose J.F., de Medeiros H.S., Bernardes P.C., de Andrade N.J. (2014). Removal of Salmonella enterica Enteritidis and Escherichia coli from green peppers and melons by ultrasound and organic acids. Int. J. Food Microbiol..

[39] Kang D., Jiang Y., Xing L., Zhou G., Zhang W. (2017). Inactivation of Escherichia coli O157:H7 and Bacillus cereus by power ultrasound during the curing processing in brining liquid and beef. Food Res. Int..

[40] Mayo D.W., Miller F.A., Hannah R.W. (2003). Course Notes on the Interpretation of Infrared and Raman Spectra.

[41] Szigedi T. (2014). Módszerfejlesztés Fourier-Transzformációs Közeli Infravörös Technika (FT-NIR) Alkalmazási Körének Kibővítése Élelmiszeripari Mintákon. Ph.D. Thesis.

[42] Stefanović A.B., Jovanović J.R., Dojčinović M.B., Lević S.M., Nedović V.A., Bugarski B.M., Knežević-Jugović Z.D. (2017). Effect of the Controlled High-Intensity Ultrasound on Improving Functionality and Structural Changes of Egg White Proteins. Food Bioprocess Technol..

[43] Xie Y., Wang J., Wang Y., Wu D., Liang D., Ye H., Cai Z., Ma M., Geng F. (2020). Effects of high-intensity ultrasonic (HIU) treatment on the functional properties and assemblage structure of egg yolk. Ultrason. Sonochem..

[44] Jovanović J.R., Stefanović A.B., Sekuljica N.Z., Jakovetić Tanasković S.M., Dojčinović M.B., Bugarski B.M., Knežević-Jugović Z.D. (2016). Ultrasound Pretreatment as an Useful Tool to Enhance Egg White Protein Hydrolysis: Kinetics, Reaction Model, and Thermodinamics. J. Food Sci..

